# A pilot study of durvalumab and tremelimumab and immunogenomic dynamics in metastatic breast cancer

**DOI:** 10.18632/oncotarget.24867

**Published:** 2018-04-10

**Authors:** Cesar August Santa-Maria, Taigo Kato, Jae-Hyun Park, Kazuma Kiyotani, Alfred Rademaker, Ami N. Shah, Leeaht Gross, Luis Z. Blanco, Sarika Jain, Lisa Flaum, Claudia Tellez, Regina Stein, Regina Uthe, William J. Gradishar, Massimo Cristofanilli, Yusuke Nakamura, Francis J. Giles

**Affiliations:** ^1^ Northwestern University, Robert H. Lurie Comprehensive Cancer Center, Chicago, Illinois, USA; ^2^ The University of Chicago, Department of Medicine, Chicago, Illinois, USA; ^3^ The University of Chicago, Department of Surgery, Chicago, Illinois, USA; ^4^ Johns Hopkins, Sidney Kimmel Comprehensive Cancer Center, Baltimore, Maryland, USA

**Keywords:** metastatic breast cancer, immune checkpoint, T cell receptor sequencing, immunogenomics

## Abstract

Immune checkpoint inhibitors produce modest responses in metastatic breast cancer, however, combination approaches may improve responses. A single arm pilot study was designed to determine the overall response rate (ORR) of durvalumab and tremelimumab, and evaluate immunogenomic dynamics in metastatic endocrine receptor (ER) positive or triple negative breast cancer (TNBC). Simon two-stage design indicated at least four responses from the first 18 patients were needed to proceed with the second stage. T-cell receptor (TCR) sequencing and immune-gene expression profiling were conducted at baseline and two months, whole exome sequencing was conducted at baseline. Eighteen evaluable patients were accrued (11 ER-positive; seven TNBC). Only three patients had a response (ORR = 17%), thus the study did not proceed to the second stage. Responses were only observed in patients with TNBC (ORR = 43%). Responders versus non-responders had upregulation of *CD8*, *granzyme A*, and *perforin 1* gene expression, and higher mutational and neoantigen burden. Patients with TNBC had an oligoclonal shift of the most abundant TCR-beta clonotypes compared to those with ER-positive disease, *p* = 0.004. We conclude responses are low in unselected metastatic breast cancer, however, higher rates of clinical benefit were observed in TNBC. Immunogenomic dynamics may help identify phenotypes most likely to respond to immunotherapy.

## INTRODUCTION

The immune system can be harnessed to detect and destroy cancer cells through inhibition of immune checkpoints, such as cytotoxic T-lymphocyte associated protein 4 (CTLA-4) and programmed cell death 1 or its ligand (PD-1 and PD-L1) [1]. Early phase studies of PD-1 or PD-L1 antibodies in heavily pre-treated triple negative breast cancer (TNBC) patients demonstrated response rates of 18–24% whereas endocrine receptor (ER)–positive breast cancer has shown response rates of 12% in patients with tumors expressing PD-L1 [2–5]. Inhibition of both CTLA-4 and the PD-1/PD-L1 axis has demonstrated clinical benefit in several tumor types [6, 7].

Immunogenomics is the study of the genetic characterization of immune and cancer cell interactions [8, 9]. Tumor neoantigens are generated by somatic genomic alterations and are important for the adaptive immune system to recognize cancer cells. A greater mutational load may correlate with an increased number of neoantigens, increasing the chances of having an immunogenic neoantigen. Diverse neoantigen landscapes are more likely to stimulate neoantigen-specific T cells expansion. T cell receptor (TCR) diversity can be quantified by next generation sequencing platforms [10–14]. In patients with metastatic melanoma treated with nivolumab, a PD-1 inhibitor, TCR repertoire analysis revealed oligoclonal expansion in the tumor tissues of patients who demonstrated response [13]. These data suggest clonal T cell expansion, determined by TCR sequencing, may be an important predictive biomarker to immunotherapy. Thus, while there are numerous assays to investigate immune-related biomarkers, we chose to focus on these immunogenomic biomarkers. We hypothesized that treatment with durvalumab and tremelimumab in patients with refractory metastatic breast cancer would produce clinical responses, and immunogenomic biomarkers would be associated with response to therapy.

## RESULTS

### Patients

**Figure 1:**
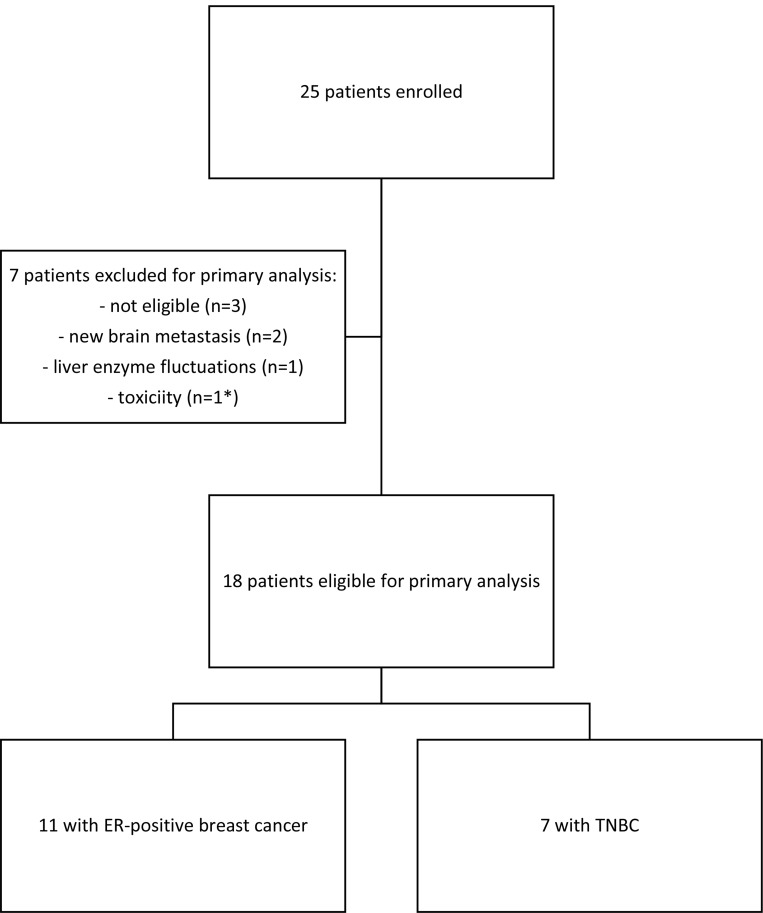
CONSORT diagram

From January until September 2016, a total of 25 patients were enrolled; only 18 patients were eligible for analysis, since seven did not reach the two-month endpoint (Figure 1). Table 1 reviews cohort characteristics.

**Table 1:** Patient characteristics of the entire cohort and by breast cancer subtype (TNBC and ER-positive)

	All (*n* = 18)	ER-positive (*n* = 11)	TNBC (*n* = 7)	*p*-value
Age, mean (range)	57.2 (38–83)	58.0 (40–83)	55.9 (38–77)	
Race, n (%)				
CA	16 (89%)	10 (91%)	6 (86%)	0.99
AA	2 (11%)	1 (9%)	1 (14%)	
Ethnicity				
Hispanic	3 (17%)	1 (9%)	2 (29%)	0.53
Non-Hispanic	15 (83%)	10 (91%)	5 (71%)	
ECOG				
0	7 (39%)	5 (45%)	2 (29%)	0.64
1	11 (61%)	6 (55%)	5 (71%)	
Metastatic site				
Bone	11 (61%)	9 (82%)	2 (29%)	0.049
Liver	12 (67%)	9 (82%)	3 (43%)	0.14
Lung	7 (37%)	5 (45%)	2 (29%)	0.64
Lymph nodes	9 (50%)	2 (18%)	7 (100%)	0.002
Other	5 (28%)	2 (18%)	3 (43%)	0.33
Metastatic site biopsied				
Bone	1 (6%)	1 (9%)	0 (0%)	0.034
Liver	8 (44%)	7 (64%)	1 (14%)	
Lymph nodes	2 (11%)	0 (0%)	2 (29%)	
Other	7 (39%)	3 (27%)	4 (57%)	
Number of prior therapies in metastatic setting				
1–2	8 (44%)	4 (36%)	4 (57%)	0.82
3–5	3 (17%)	2 (18%)	1 (14%)	
5+	7 (37%)	5 (45%)	2 (29%)	
Previous chemotherapy in metastatic setting				
Taxanes	15 (83%)	9 (82%)	6 (86%)	0.99
Anthracycline	14 (78%)	9 (82%)	5 (71%)	0.99
Platinum	9 (50%)	3 (27%)	6 (86%)	0.05

### Efficacy and safety

**Figure 2:**
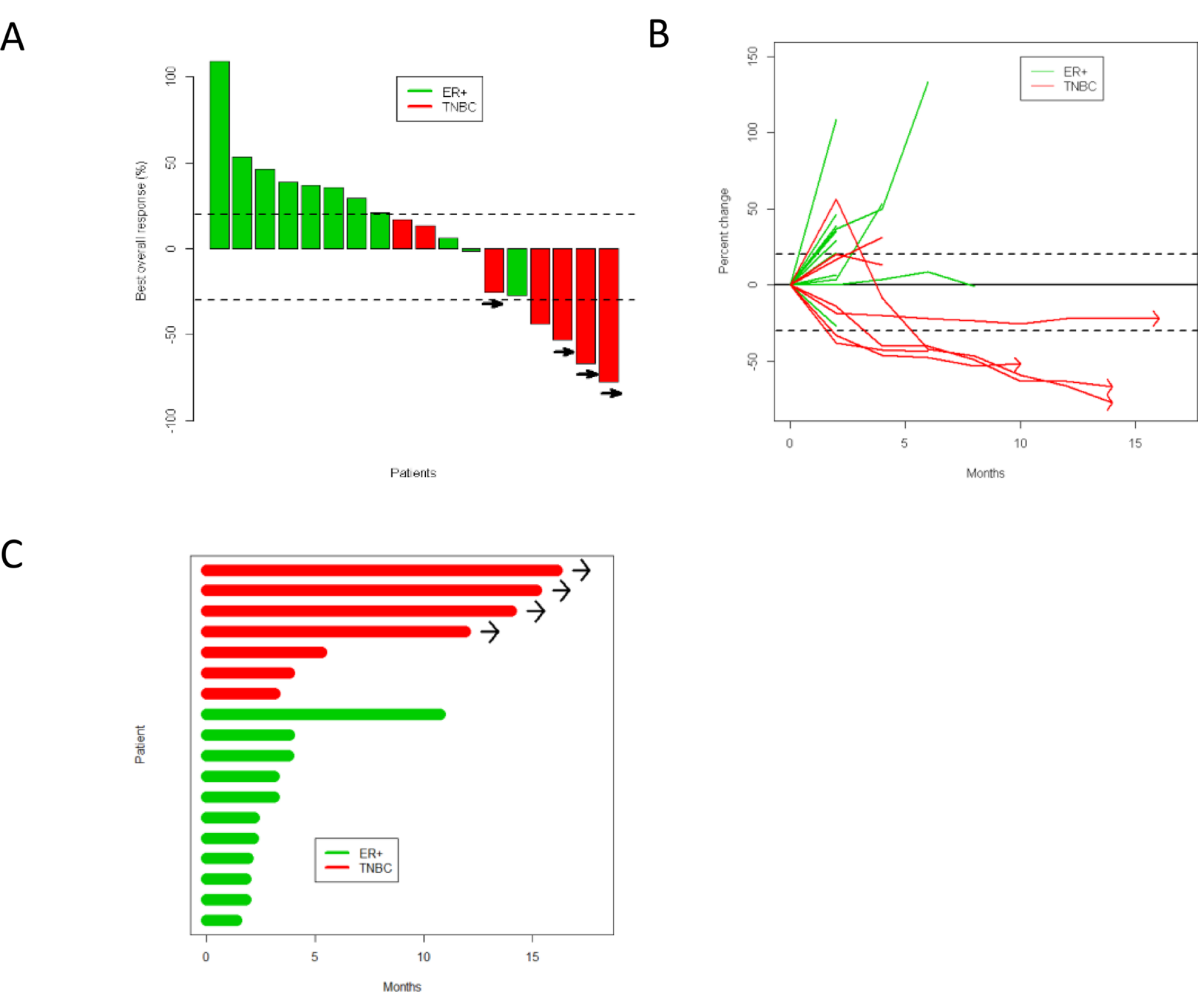
Response to durvalumab and tremelimumab shown by (**A**) waterfall plot, (**B**) spider plot, and (**C**) swimmers plot. Green is ER-positive, red is TNBC. Patients with continued clinical benefit beyond data cutoff date are identified by arrows.

Three partial responses by Response Evaluation Criteria In Solid Tumors (RECIST) were noted among these 18 patients, and the study was suspended as it did not meet Simon two-stage criteria to move onto the next stage. The overall response rate (ORR) of the entire cohort was 17%, 0% in ER-positive patients, and 43% in TNBC (Supplementary Table 1). At the time of data cut-off and analysis, four patients with TNBC had sustained responses for 10 months or longer (Figure 2). Notably, one patient with TNBC was noted to have PD per RECIST because of a lymph node which enlarged by two months; however, all target lesions responded and the lymph node which had enlarged remained stable; this patient did not contribute to the reported ORR. Patients who had sustained responses generally had lower visceral disease burden (only one of the four patients had liver metastasis); and had only received one line of cytotoxic chemotherapy in the metastatic setting.

**Figure 3:**
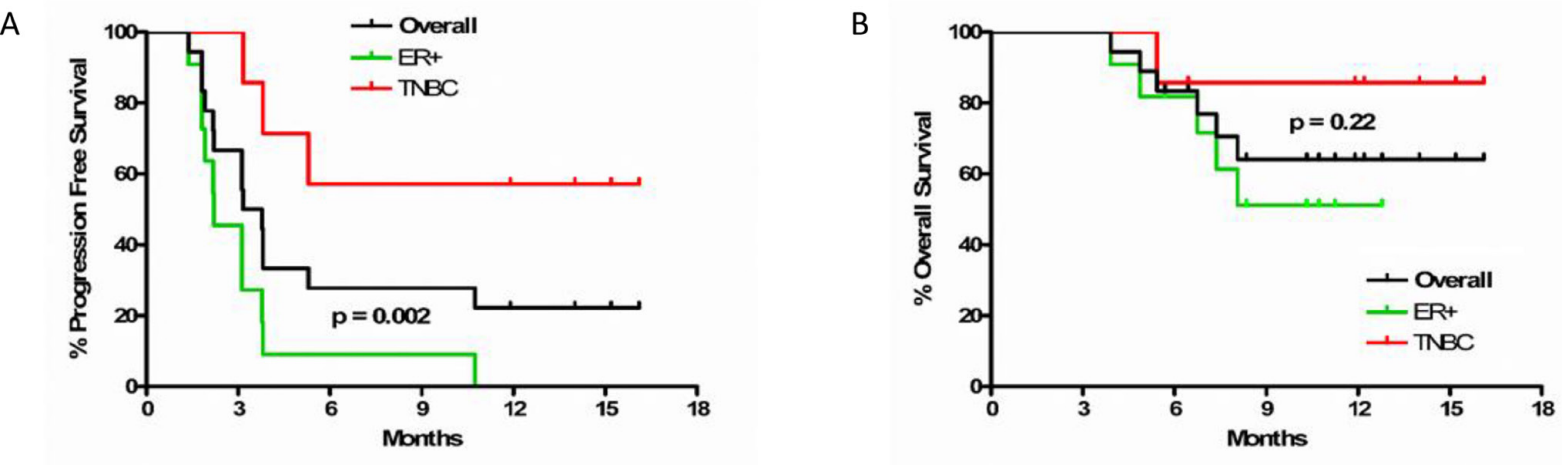
(**A**) Progression free and (**B**) overall survival in overall cohort and by subtype of breast cancer. Dark grey line is overall cohort, green is ER-positive, and red is TNBC.

Median PFS in the TNBC cohort was not reached, and was 2.2 months (95% confidence interval (CI) 1.8–3.8) in the ER-positive cohort (hazard ratio (HR) = 6.4, 95% CI 1.7–24.3, *p* = 0.002). Median OS was not reached in the TNBC or ER-positive cohorts (HR = 3.6, 95% CI 0.4–30.6, *p* = 0.22) (Figure 3). Hepatitis, electrolyte abnormalities, and rash were the most common related adverse events. No grade 4 or 5 treatment-related adverse events were observed. Grade 3 adverse events or those with a frequency of greater than 10 are summarized in Supplementary Table 2.

### Immunogenomic dynamics

#### Non-synonymous mutation load was higher in responders

**Figure 4:**
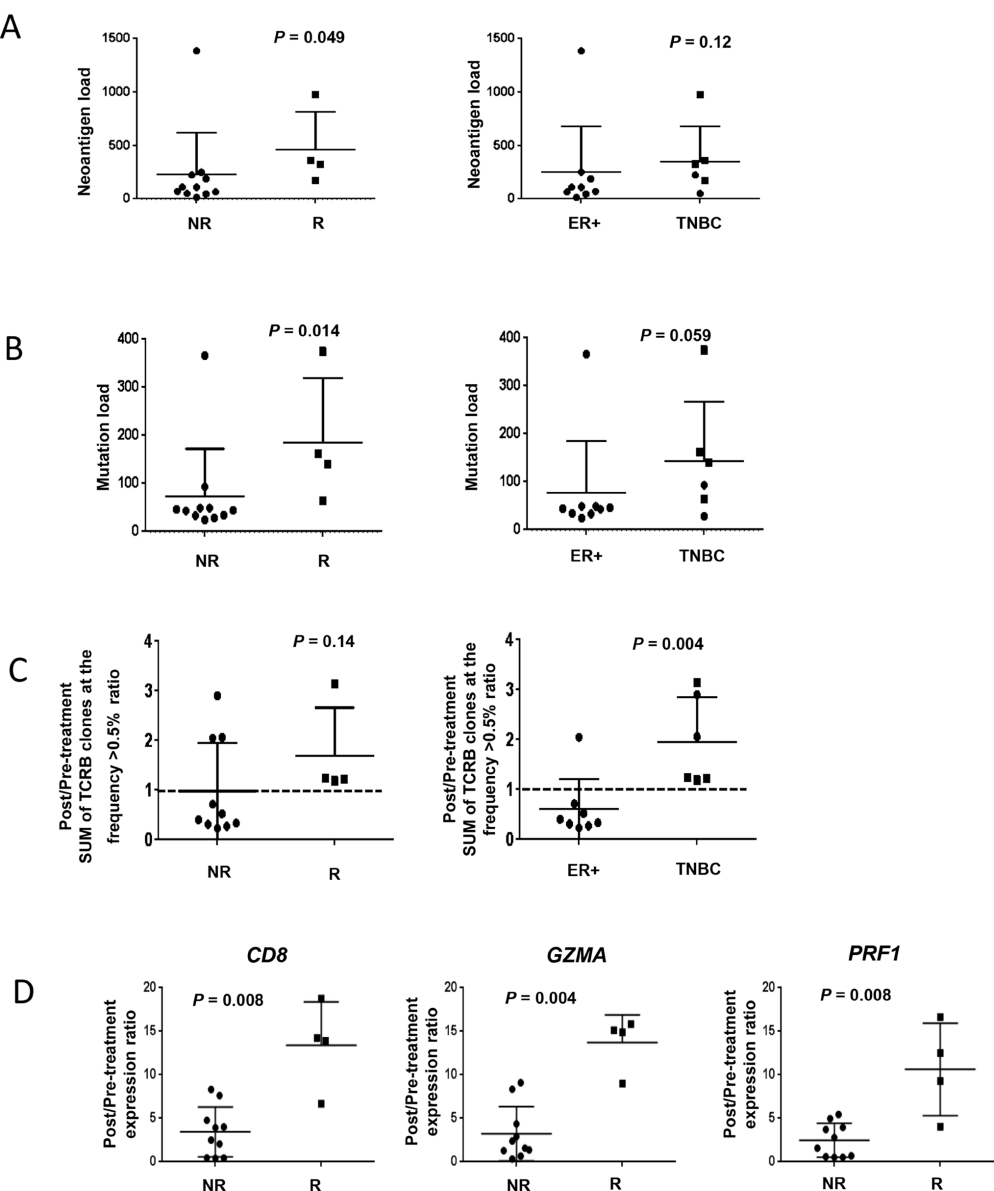
Association of immunogenomic biomarkers and breast cancer subtype and response Circular and square dots indicate non-responders (NR) and responders (R), respectively. (**A**) The numbers of non-synonymous mutations and (**B**) predicted neoantigen epitopes were significantly higher in responders, and tended to be numerically higher in TNBC subtype although not significant. (**C**)The total proportion of the abundant TCRB CDR3 clonotypes with the frequency of 0.5% or higher was numerically higher in responders but not statistically significant (*p* = 0.14) but was increased in TNBC (*p* = 0.004). (**D**) Correlation of baseline transcriptional levels of immune-related genes in comparison to response. (**E**) Significant increase of transcriptional levels of CD8, GZMA and PRF1 according to response.

In patients who had a response, the number of non-synonymous somatic mutations was significantly higher compared to non-responders (*p* = 0.014, Figure 4A). Furthermore, responders had significantly higher numbers of predicted neoantigens compared to non-responders (*p* = 0.049, Figure 4B). TNBC showed a tendency of higher mutation load and predicted neoantigens than ER-positive breast cancer.

#### Clonal T-cell expansion in responders to dual blockade of PD-L1 and CTLA-4

TCRB sequencing was conducted on baseline and two-month samples obtained from 14 patients (eight ER-positive and six TNBC) who had sufficiently evaluable tissue at both time points. Variable distribution patterns of TCR clonotypes were observed (Supplementary Figure 1). Through cDNA sequencing of TCRB, unique TCRB complementarity determining region 3 (CDR3) clonotypes of 28,595 ± 26,864 were identified in individual tissues (Supplementary Table 3). After sorting out CDR3 clonotypes according to their frequencies in tumor tissue samples, proportions of abundant CDR3 clonotypes (defined by frequency of ≥0.5%) were counted in each tumor tissue, and the sum of the abundant CDR3 clonotypes was found to be increased by treatment in seven of 14 cases (Supplementary Figure 1). Furthermore, the post/pre-treatment ratio was calculated for each patient according to the sum of abundant TCRB CDR3 clonotypes. As a result, the sum of abundant TCRB CDR3 clonotypes was increased in all responding patients although not statistically significant (*p* = 0.14, Figure 4C). Interestingly, according to breast cancer subtype, the sum of abundant TCRB CDR3 clonotypes was significantly increased in TNBC tumors compared to ER-positive tumors (*p* = 0.004, Figure 4C), suggesting that TNBC may have the unique tumor microenvironment where a subset of T cells could be efficiently expanded after dual blockade of PD-L1 and CTLA-4. A correlation between TCRB changes and neoantigen load was not observed (Supplementary Figure 2).

#### Cytolytic immune-related genes are upregulated after dual blockade

Baseline levels of immune-related genes were not significantly different between responders versus non-responders (Supplementary Figure 3). However, responders had an increase in *CD8*, *GZMA* and *PRF1* expression levels compared to non-responders (Figure 4D), implying that cytolytic T lymphocytes with high cytotoxic activity were strongly infiltrated, expanded, and activated in cancer tissues by dual blockade in responders and TNBC patients.

#### Plasma cell infiltration in pseudoprogression

**Figure 5:**
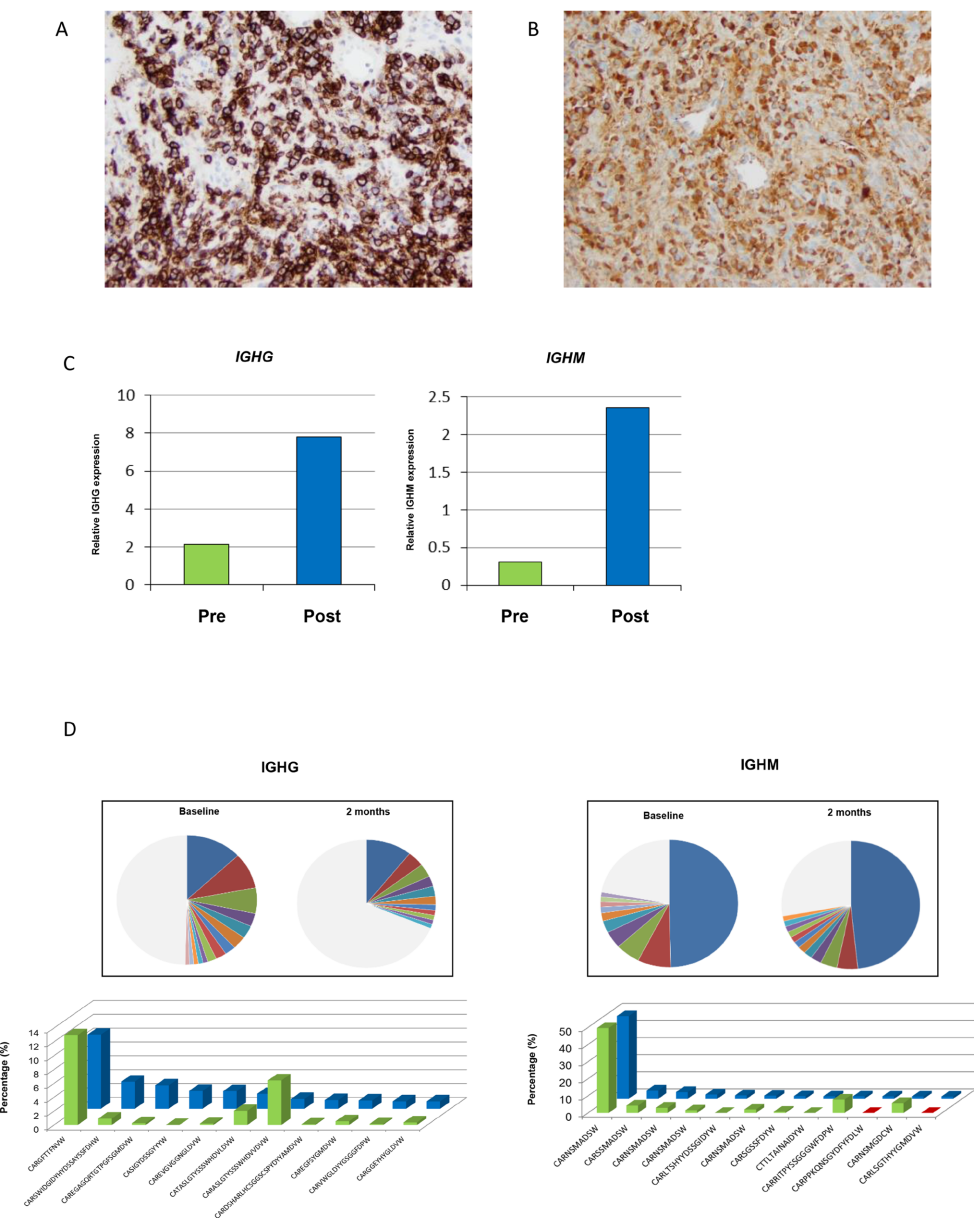
Exploratory characterization of B-cells in patient with pseudoprogression **(A)** Immunohistochemical confirmation of presence of plasma cells expressing CD138. (**B**) Further analysis demonstrates intense IgG immunohistochemical staining. (**C**) Gene expression of IGHG and IGHM, and (**D**) changes in abundant BCR clones (frequency ≥1.0%) for IGHG and IGHM (green is baseline, and blue is at two months).

As noted in Figure 2B, one patient had an increase in their tumor by over 50% before subsequent decrease. Per protocol the patient was biopsied at two months, which was the height of the radiographic progression. A hematoxylin and eosin stained slide was prepared, and a dense cluster of cells with an eccentric nuclei, perinuclear hoffs, and clockface chromatin characteristic of plasma cells were observed at the tumor periphery. Immunohistochemistry (IHC) analysis demonstrated strong CD138 expression, confirming plasma cell infiltration (Figure 5A). IHC staining further revealed high levels of IgG immunoreactivity (Figure 5B). To characterize the immunoglobulin heavy chain (IGH) isotypes in these plasma cells, transcriptional levels of *IGHG, IGHM, IGHA* and *IGHD* using real-time RT-PCR method was performed. Increased expression levels of all isotype genes in the post-treatment tumor was noted, although *IGHG* and *IGHM* predominated (Figure 5C, Supplementary Figure 4). To further characterize the clonality of these tumor-infiltrated plasma cells, B cell receptor (BCR) sequencing of pre- and post-treatment tumor tissues was performed. Most of the enriched CDR3 clonotypes (defined by frequency of ≥1%) in the post-treatment tumor were either rarely present or absent in the pre-treatment tumor (Figure 5D).

## DISCUSSION

This is the first study published evaluating dual checkpoint blockade and immunogenomic dynamics in metastatic breast cancer. Limitations of the study include a small sample size, and single arm design; however, while not definitive, these data provide several important observations that merit further study.

Toxicity observed was consistent with those known to durvalumab and tremelimumab and previous reports. As with experience with single agent PD-1/PD-L1 blockade, responses are higher in TNBC than in ER-positive disease. Additional studies are required to further assess the role of combination checkpoint blockade in TNBC, and identify subgroups that are more susceptible to PD-L1 and CTLA-4 inhibition. We observed that the responders had low burden of metastatic disease, and in fact, their disease was predominantly restricted to lymph nodes. Consistent with recently reported studies, patients who experienced sustained responses were less heavily pretreated (only one line of cytotoxic chemotherapy in the metastatic setting) [15, 16]. We noted two cases of pseudoprogression, one which per RECIST counted as PD and did not contribute to the reported ORR despite the patient experiencing over one year of disease control. Future studies using immune checkpoint inhibitors should consider immune related RECIST or other endpoints in their design so that cases of pseudoprogression and clinical benefit can be accounted for.

This is the first study to comprehensively investigate immunogenomic profiling in patients with metastatic breast cancer receiving immune checkpoint blockade. Although our sample size is small, we demonstrate a trend that tumors with higher numbers of non-synonymous somatic mutations and predicted neoantigens are associated with response, consistent with other published data [17, 18]. Higher non-synonymous mutation burden may increase the likelihood of generating immunogenic mutated peptides, which may in turn activate antigen-specific T cells, although, in this small study we did not observe a significant correlation between mutational load and the sum of the abundant CDR3 clonotypes (Supplementary Figure 2). Patients with TNBC who responded had a small subset of T cells significantly expanded in tumor tissues after immunotherapy, suggesting that this treatment regimen strongly stimulated local immune response and likely induce activated T cells to recognize cancer-specific antigens. Indeed, we detected that *CD8*, *GZMA* and *PRF1* transcription levels were drastically increased in post-treatment tumor tissues of patients who responded, suggesting an activated and expanded T cell repertoire. Identifying markers of early response is crucial in developing immunotherapy as response may occur after several months of therapy and radiographic increase in tumors may be misdiagnosed as pseudoprogression [2, 19].

We observed a dense cluster of plasma cells at the tumor periphery in a patient with pseudoprogression who went on to have long-term disease control. There was upregulation of all IGH isotypes, and furthermore, new BCR clonotypes were observed, suggesting infiltrating plasma cells may augment the local immune response along with cytotoxic T cells by producing specific antibodies to CDR3 clonotypes. It is known that plasma cells play a critical role in generating highly diverse antibody repertoires in the tumor microenvironment to secrete antibodies against endogenous tumor-associated antigens, and that there is significant crosstalk with T cells [20]. These findings suggest that PD-L1 and CTLA-4 blockade may affect crosstalk between T cells and B cells, possibly inducing plasma cells to produce specific antibodies in the tumor microenvironment. Future studies investigating the role of plasma cells in patients with durable responses are warranted, as identification of cancer-specific antibodies may lead to novel personalized immunotherapeutic approaches.

In summary, combination durvalumab and tremelimumab has low response rates in unselected metastatic breast cancer; however, patients with TNBC may be more likely to benefit. We observed response rates significantly higher that those reported with single agent PD-1/PD-L1 inhibition in metastatic TNBC; however, these results require validation. Immunogenomic biomarkers were associated with responders and TNBC, and will be validated in an ongoing study (NCT02536794). Development of predictive biomarkers of response are critical in developing immunotherapeutic strategies for breast cancer.

## MATERIALS AND METHODS

### Patient population

Eligible patients had stage IV histologically documented human epidermal growth factor 2-neu (HER2) negative breast cancer and had received at least one line of chemotherapy. A total of 28 patients were planned to be enrolled, 14 with TNBC and 14 with ER-positive breast cancer. Patients with ER-positive breast cancer must have progressed through endocrine therapy and be considered endocrine refractory.

### Study design

The protocol was approved by the institutional review board at Northwestern University and complied with the International Ethical Guidelines for Biomedical Research Involving Human Subjects, good clinical practice guidelines, the Declaration of Helsinki, and local laws. All subjects provided written informed consent. This was a single arm, open-label, pilot study designed to determine the efficacy of durvalumab and tremelimumab in patients with metastatic HER2-negative breast cancer, and relationship of response to immunogenomic dynamics. The primary objective of clinical efficacy was assessed by the overall response rate, defined as patients with a partial response (PR) or complete response (CR) by RECIST version 1.1 criteria . Secondary objectives were to assess progression free survival (PFS) and overall survival (OS), and the safety and tolerability of durvalumab and tremelimumab in metastatic breast cancer. Safety and tolerability was assessed by the number, frequency, and severity of adverse events as defined by the Common Terminology Criteria for Adverse Events (CTCAE) version 4.03. Immunogenomic biomarkers evaluated included TCR sequencing, whole exome sequencing to establish mutational and neoantigen burden, and immune-related gene expression profiling.

### Treatment protocol and procedures

Patients were treated until progressive disease or unacceptable toxicity with durvalumab 1500mg IV and tremelimumab 75mg IV monthly for four doses, followed by durvalumab 750mg reevery two weeks to complete one year of therapy, with the option to renew therapy for an additional year if patients were deriving clinical benefit. Eligible patients must have completed two months of therapy to be included in the analysis of the primary objective; however, all patients who received at least one dose of treatment were included in toxicity analysis. Patients with radiographic progression without clinical signs or symptoms indicating progression or decline in functional status were eligible to continue to receive treatment on protocol. Tumor biopsies and collection of peripheral blood were obtained at baseline and two months after starting treatment. Tumor biopsies were collected from radiographically accessible metastatic sites, and the same site sampled at baseline was again sampled at two months.

### Correlative studies and molecular analysis

#### Mutational and neoantigen burden

Genomic DNA and total RNA were extracted from tumor biopsies using *AllPrep* DNA/RNA mini kit (Qiagen, Valencia, CA). As germline control DNA, genomic DNA was extracted from peripheral blood mononuclear cells (PBMCs). Whole-exome libraries were prepared from 1,000 ng of genomic DNAs using SureSelectXT Human All Exon V5 kit (Agilent Technologies, Santa Clara, CA) and the prepared whole-exome libraries were sequenced by 100-bp paired-end reads on HiSeq2500 Sequencer (Illumina, San Diego, CA).

Sequence alignment and mutation calling were performed using our in-house pipelines described previously [21]. Briefly, the sequence reads were mapped to the human reference genome GRCh37/hg19 using Burrows-Wheeler Aligner (BWA) (v0.7.10) [22]. Possible PCR duplicated reads were removed using Picard tool (http://broadinstitute.github.io/picard/), and read with a mapping quality of <30 and with mismatches of more than 5% of nucleotides were also excluded. Finally, somatic variants (single nucleotide variations (SNVs) and indels) were called with the following parameters, (i) base quality of ≥15, (ii) sequence depth of ≥10, (iii) variant depth of ≥2, (iv) variant frequency in tumor of ≥10%, (v) variant frequency in normal of <2%, and (vi) Fisher *p* value of <0.05.

Using whole-exome sequence data of the germline DNAs, HLA class I genotypes of each patient were determined by OptiType algorithm [23]. Subsequently, neoantigens for each HLA allele were predicted from non-synonymous mutations identified through the whole exome sequence data of 14 tumor samples. All 8- to 11-mer peptides harboring each substituted amino acid were investigated by applying the filtering with the predicted binding affinity to HLA-A, HLA-B and HLA-C of <500 nM, using NetMHCv3.4 and NetMHCpanv2.8 software [24–26].

#### TCR repertoire analysis

Total RNA from tumor tissues were isolated using AllPrep DNA/RNA Mini kit (Qiagen, Valencia, CA). Sequencing libraries of TCR-beta (TCRB) were prepared as described previously and subjected to sequencing on the Illumina Miseq platform, using 600 cycles Miseq Reagent Kit V3 (Illumina) [24, 27]. TCR repertories were analyzed using Tcrip software [27]. Briefly, to identify V, D, J and C segments in individual TCRB sequencing reads, each of the sequence reads in FASTQ files were mapped to the reference sequences provided by IMGT/GENE-DB [28] using Bowtie2 aligner (Version 2.1.0) [29, 30].

### BCR repertoire analysis

A similar approach to that of TCR sequencing (5′ RACE approach) was taken in preparation of a BCR library. The library pooling was subjected to sequencing on the Illumina Miseq platform, using 600 cycles Miseq Reagent Kit V3 (Illumina). To analyze BCR sequencing data, we developed an algorithm of V(D)J decomposition with soft-clipping, Bcrip software (Nakamura lab), similar to Tcrip software developed for TCR repertoire analysis.

### Gene expression analysis

Expression levels of immune-related genes were examined using quantitative RT-PCR at baseline and two-month biopsies. cDNA was synthesized from RNA using Superscript III first-strand synthesis kit (Invitrogen, Carlsbad, CA) and RT-PCR was performed in the ABI ViiA 7 system (Applied Biosystems, Foster City, CA), according to the manufacturer’s instructions. The TaqMan probes for *CD3*, *CD4*, *CD8*, *FOXP3*, *PD-L1*, *PD-L2*, *GZMA*, *PRF1*, *HLA-A*, genes were purchased from Life Technologies (Supplementary Table 4). To quantify the mRNA expression levels of each BCR isotype (*IGHG*, *IGHM*, *IGHA* and *IGHD*), we designed specific primers corresponding to parts of constant exon of each BCR as shown in Supplementary Table 5. The expression levels were normalized with that of *GAPDH*.

### Immunohistochemical analysis

Paraffin-embedded tissues were cut into 5-μm-thick sections. Each section was stained with hematoxylin-eosin stain according to standard protocols. Immunohistochemistry was performed using a CD138 (Syndecan 1) antibody (MI15, Leica Biosystems, Buffalo Grove, IL) and IgG antibody (RWP49, Leica Biosystems, Buffalo Grove, IL).

### Statistical analysis

#### Sample size and design

Using a Simon’s two-stage design, we assumed the undesirable ORR (null hypothesis) to be approximately 10%, and the alternate hypothesis to be approximately 30% based on previously published and presented data [2]. Eighteen patients were planned in the first stage. If four or more responded, then an additional 10 patients were planned to be added for a total of 28. This design has a Type I error rate of 4% and 80% power, and has a 90% chance of stopping early after the first stage if the true response rate is 10%. If the study did not progress onto the second stage, and only 18 patients were enrolled, there was 78% power to detect the 10% versus 30% difference if 5 or more of 18 patients responded. The design was based on the complete sample, not stratified by ER status. Although subgroup analysis by subtype was anticipated it is not powered for definitive conclusions and thus exploratory in nature.

#### Endpoint analysis

The primary analysis of ORR was based on all patients evaluable for response. ORR was defined as PR or CR by RECIST, maximum response prior to disease progression was used. The ORR was estimated by the proportion of overall response, and its 80% confidence interval (CI) and 95% CI was estimated using the exact binomial distribution. Subgroup analysis was planned to be calculated separately by receptor status, but the study was not powered for analyses within these subgroups. Secondary endpoints of PFS and OS were estimated using the Kaplan-Meier and Cox proportional hazards methods. PFS was calculated as date of enrollment until date of progression in the absence of clinical benefit or continued therapy to account for patients with pseudoprogression. The number, frequency, and severity of adverse events as defined by CTCAE version will be recorded and summarized. The Mann-Whitney *U* test (two-tailed) was performed for comparison of baseline and two-month ratios of total proportions of the most abundant CDR3 clonotypes (frequency of ≥0.5%), non-synonymous mutation load, neoantigens load, gene expression levels between non-responders and responders (defined as SD > 6months or CR) patients, or breast cancers subtype (ER-positive and TNBC). Statistical analyses were done using GraphPad Prism version 6.0 (GraphPad software, La Jolla, CA) and SAS (SAS Institute Inc. 2012. SAS OnlineDoc^®^ 9.4. Cary, NC: SAS Institute Inc.). A *p* value of < 0.05 was considered statistically significant.

## SUPPLEMENTARY MATERIALS FIGURES AND TABLES



## Abbreviations

PD-1: Programed cell death 1
PD-L1: Programmed cell death ligand 1
CTLA-4: Cytotoxic T-lymphocyte associated protein 4
TNBC: Triple negative breast cancer
ER: Endocrine receptor
HER2: Human epidermal growth factor 2-neu
IHC: Immunohistochemistry
TCR: T cell receptor
RECIST: Response Evaluation Criteria In Solid Tumors
CTCAE: Common Terminology Criteria for Adverse Events
ORR: Overall response rate
SD: Stable disease
PR: Partial response
PD: Progressive disease
PFS: Progression free survival
OS: Overall survival
PBMCs: Peripheral blood mononuclear cells
BWA: Burrows-Wheeler Aligner
