# Supplementary material for: A pilot study of durvalumab and tremelimumab and immunogenomic dynamics in metastatic breast cancer
Source: Oncotarget. 2018 Apr 10;9(27):18985–96. doi: 10.18632/oncotarget.24867 (PMC5922371; doi:10.18632/oncotarget.24867)
Supplement: Supplementary file 1 [file oncotarget-09-18985-s001.pdf]

A pilot study of durvalumab and tremelimumab and immunogenomic dynamics in metastatic breast cancer

SUPPLEMENTARY MATERIALS

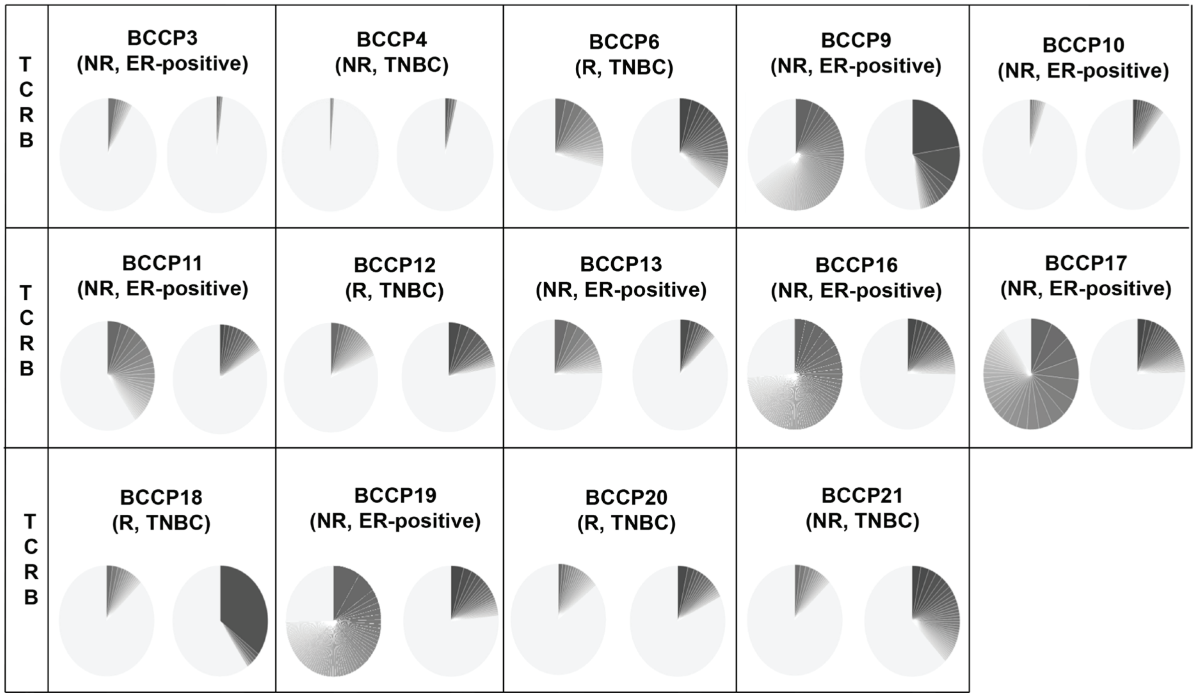

Supplementary Figure 1: The distribution of the abundant TCRB CDR3 clonotypes (frequency ≥ 0.5%) is presented in pie charts (baseline and at two months, respectively). Light grey color indicates portion of CDR3 clonotypes below the read frequency of 0.5%.

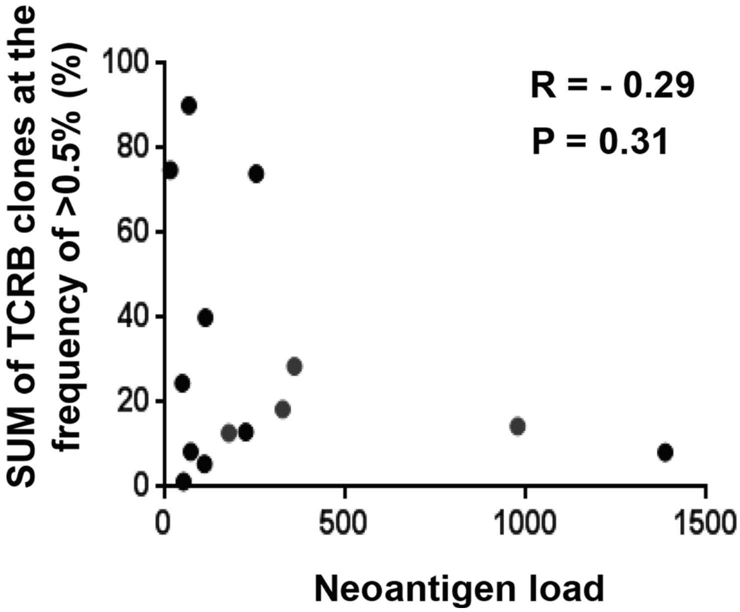

Supplementary Figure 2: Correlation between mutational load and the sum of the abundant CDR3 clonotypes.

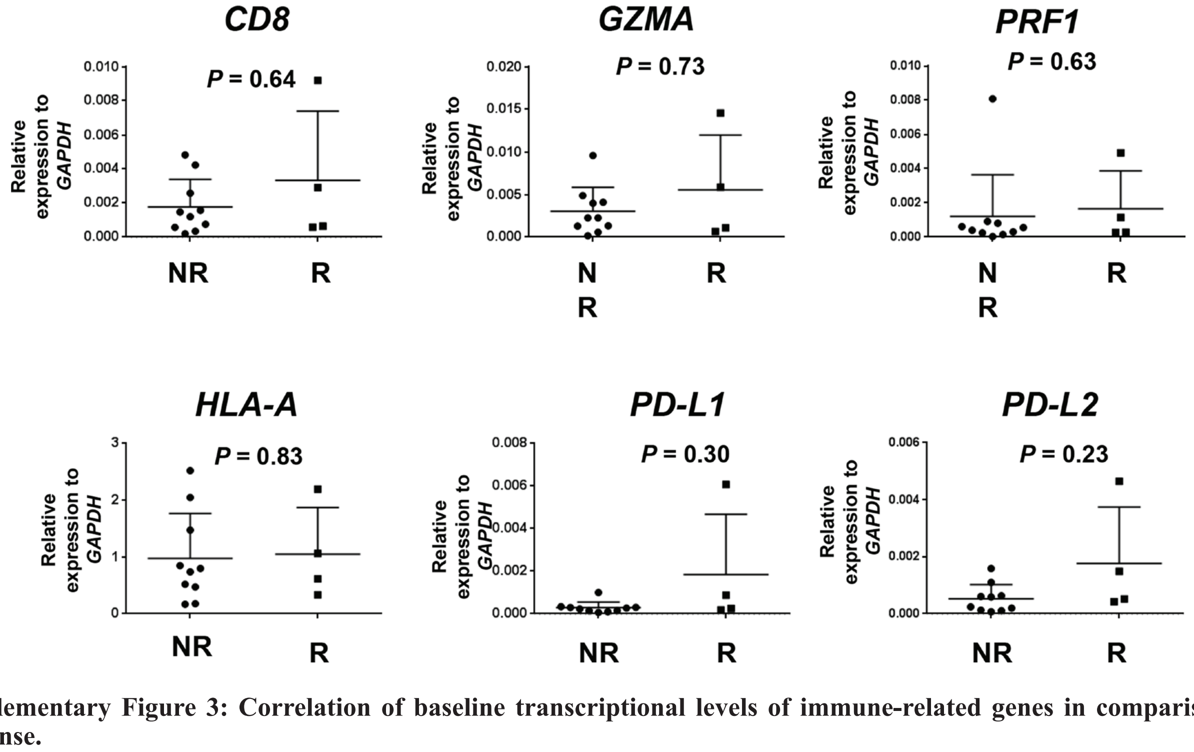

Supplementary Figure 3: Correlation of baseline transcriptional levels of immune-related genes in comparison to response.

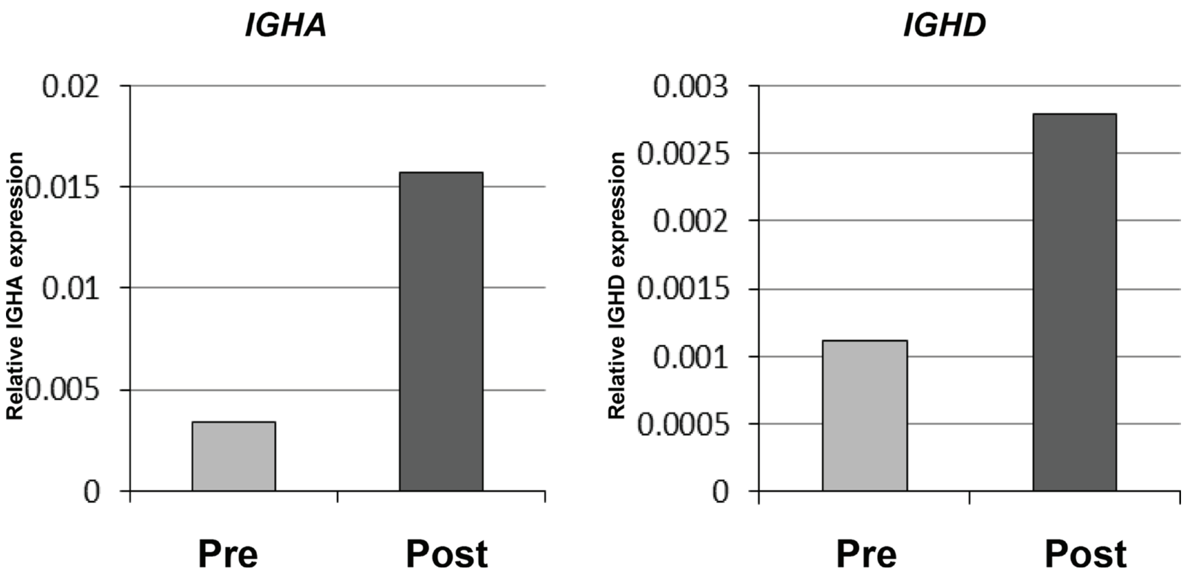

Supplementary Figure 4: Expression levels of *IGHA* and *IGHD* in patient with pseudoprogression at baseline and two months of therapy.

Supplementary Table 1: Responses according to standard RECIST criteria of the entire cohort and by subtype

	All patients (n = 18)	ER-positive (n = 11)	TNBC (n = 7)
CR	0 (0%)	0 (0%)	0 (0%)
PR	3 (17%)	0 (0%)	3 (43%)
SD ≥6 months	4 (22%)	2 (19%)	1 (14%)
PD	11 (61%)	9 (82%)	*3 (43%)
ORR	3 (17%)	0 (0%)	3 (43%) [irORR 4 (57%)]
CBR	7 (39%)	2 (19%)	4 (57%) [irCBR 5 (71%)]

*includes one patient with pseudoprogression who had clinical benefit >12 months.

Supplementary Table 2: Most common treatment related adverse events or grade 3 or higher

	Grade 1/2	Grade 3	Total
Hepatitis	14	3	17
Electrolyte abnormalities	11	1	12
Rash and skin condition	12	0	12
Glucose abnormality	10	1	11
Fatigue	10	0	10
Anemia	6	2	8
Alkaline phosphatase/GGT increased	6	2	8
Hypoalbuminemia	6	1	7
Lymphocyte count decreased	3	1	4
Blood bilirubin increased	3	1	4
Arrhythmia/palpitations	2	1	3
Pain	2	1	3
Creatinine increased	2	1	3
Dyspnea	1	1	2
Myocarditis	0	1	1

Supplementary Table 3: Summary of TCRB sequence results

	Pre-treatment			Post-treatment		
	Total Reads	Observed Clonotypes	Unique Clonotypes	Total Reads	Observed Clonotypes	Unique Clonotypes
BCCP3	278255	191230	46748	145248	89668	32193
BCCP4	1018949	120800	52274	1068868	91745	49664
BCCP6	2151676	87220	7803	1597554	135864	7142
BCCP9	1005213	20357	2014	725329	253787	14898
BCCP10	1605373	1319043	70535	766064	593005	29127
BCCP11	1290282	508063	18966	1313932	998358	42060
BCCP12	1175944	903595	93840	843311	686756	54673
BCCP13	487720	361207	23066	1196537	936186	61184
BCCP16	653287	565773	12990	280544	203558	8356
BCCP17	757788	400333	11012	496704	394476	16297
BCCP18	638880	435490	26672	507161	360006	18330
BCCP19	961971	823547	17220	323897	223181	13724
BCCP20	501746	424632	37015	524611	396309	41263
BCCP21	2517801	120912	6256	79687	58058	6547

Supplementary Table 4: List of Taqman probes designed for quantitative RT- PCR of immune-related genes

Marker	Gene	Assay ID
CD3	*CD3E*	Hs01062241_m1
CD4	*CD4*	Hs01058407_m1
CD8	*CD8A*	Hs002335520_m1
FOXP3	*FOXP3*	Hs01085834_m1
Perforin 1	*PRF1*	Hs00169473_m1
Granzyme A	*GZMA*	Hs00989184_m1
HLA-A	*HLA-A*	Hs01058806_g1
PD-L1	*CD274*	Hs01125301_m1
PD-L2	*PDCD1LG2*	Hs01057777_m1
GAPDH	*GAPDH*	Hs02758991_g1

Supplementary Table 5: Primer sequences for quantitative RT- PCR of *IGH* genes

Primer	Sequence (5′ to 3′)
qIGHG-Forward	GCCTGGTCAAGGACTACTTC
qIGHG-Reverse	CACGCTGCTGAGGGAGTAG
qIGHM-Forward	ACCATCAAAGAGAGCGACTG
qIGHM-Reverse	CGGATGGCTGTGTCTTGATC
qIGHA-Forward	AGTACCTGACTTGGGCATCC
qIGHA-Reverse	CCCACCATGCAGGAGAAGG
qIGHD-Forward	GGAACAAGAAGAGAGAGAGAC
qIGHD-Reverse	AGCAGGTGAAGGTGGCTTTG
